# Testing Reliability of Biophilic Design Matrix Within Urban Residential Playrooms

**DOI:** 10.3389/fpsyg.2020.570099

**Published:** 2020-12-09

**Authors:** Ellen Marte, Abigail Calumpit, Bárbara de Sá Bessa, Ashley Toledo, Roberta Fadda, Tricia Skoler

**Affiliations:** ^1^Department of Psychology, Hunter College, New York, NY, United States; ^2^Department of Pedagogy, Psychology, Philosophy, University of Cagliari, Cagliari, Italy

**Keywords:** biophilia, biophilic design, Biophilic Interior Design Matrix, children, nature play, playroom, urban

## Abstract

Playtime in urban cities has become an indoor activity for children due to limited access to natural outdoor environments. This product of urbanization makes the case for the introduction of biophilic design. However, playrooms are often neglected as a possibility in designing a natural space indoors. Interior designers and other specialists lack a reliable tool to identify and incorporate biophilic features into the design of these indoor environments in urban settings. The Biophilic Interior Design Matrix (BID-M) developed by McGee and Marshall-Baker quantifies 52 of Kellert’s biophilic design attributes to assess their presence and absence within interior spaces. We expanded its use by testing the matrix in a new type of space, urban playrooms, and coded images of 45 children’s playrooms within Manhattan residential buildings in New York City, including assessing a larger sample and reliability rate compared to McGee and Marshall-Baker’s research. Inter-rater reliability of the overall design matrix and individual matrix items was measured with percent agreement and free-marginal multirater kappa. Reliability testing showed overall good reliability of the overall design matrix. Several matrix items had low reliability between raters. Our findings show that the BID-M needs to be modified to better assess urban interior spaces for children.

## Introduction

Biophilia describes an innate need to affiliate with nature (Wilson, 1984). Active play in the natural world during childhood fosters knowledge, cognitive growth, social-emotional growth, and overall wellbeing (Fjørtoft, 2001; Chawla, 2007; McCurdy et al., 2010). The setup of cosmopolitan areas creates an obstacle for children to play outdoors in natural environments. Children on average are now only spending 4–7 min engaging in outdoor play, compared to seven and a half hours spent indoors using technology (Rideout et al., 2010). Increased media usage indoors along with low levels of active play are contributors to negative developmental outcomes such as decreases in executive functioning, negative mental health outcomes, and increased risk for attention disorders (Sackett, 2010; McHarg et al., 2020). The need for children to experience nature has become essential for children’s cognitive functioning and wellbeing (Wells and Evans, 2003; Flouri et al., 2014; Hand et al., 2017). One possibility is to introduce nature into interior urban settings designed for children.

Introducing nature into a space goes beyond simply the placement of plants. Biophilic design strategies need to be implemented with consideration for those using the space, its location, and its function (Gillis and Gatersleben, 2015; Beatley, 2016). To assist designers and urban planners, Kellert (2008) identified six biophilic design elements, and within them attributes that can be incorporated into a given space. The attributes in Table 1 fall under six elements: (1) environmental features, (2) natural shapes and forms, (3) natural patterns and processes, (4) light and space, (5) place-based relationships, and (6) human-nature relationships. The purpose and impact of biophilic design are based on the Attention Restoration Theory (ART; Kaplan and Kaplan, 1989). ART suggests that nature has a restorative effect on our attentional capacity. Built environments have distracting and cognitively taxing stimuli that require constant direct attention to inhibit, resulting in mental fatigue. Nature contains intriguing stimuli that require our unexacting attention. Direct, indirect, or representational exposure to natural environments activates bottom-up involuntary attention, allowing top-down directed-attention abilities a chance to replenish.

**TABLE 1:** Listed are 52 biophilic design attributes that were included in the BID-M out of Kellert (2008) proposed 72.

**Environmental features**	**Natural shapes and forms**	**Natural patterns and processes**
Color Water Air Plants Animals Natural materials Views and vistas Fire	Botanical motifs Tree and columnar supports Animal motifs Shells and spirals Egg, oval, and tubular forms Arches, vaults, and domes Shapes resisting straight lines Simulation of natural features/biomorphy Geomorphology Biomimicry	Sensory variability/Information richness Age, change, and the patina of time Central focal point Patterned wholes Bounded spaces Transitional spaces Linked series and chains Integrations of parts to wholes Complementary contrasts Dynamic balance and tension Fractals Hierarchically organized ratios and scales

**Light and space**	**Place-based relationships**	**Human-nature relationships**

Natural light Filtered and diffused light Light and shadow Reflected light Light pools Warm light Light as shape and form Spaciousness Spatial variability Space as shape and form Spatial harmony Inside-outside space	Geographic connection to place Historic connection to place Ecological connection to place Cultural connection to place Indigenous materials Landscape orientation/landscape features	Prospect and refuge Order and complexity Curiosity and enticement Change and metamorphosis

*Exclusions were made from the BID-M if they did not pertain to interior attributes and were not able to be analyzed through an image of a space.*

The restoration of cognitive functioning when exposed to nature is found to be evident in children. Multiple studies have found that children have improved concentration and milder attention deficit symptoms as a result of nature exposure compared to exposure to urban settings (see also Taylor and Kuo, 2004, 2009, 2011). Given the expansive benefits that a connection with the natural environment has to offer both indoors and outdoors, it is imperative to provide more opportunities for children to be exposed to nature.

While the developmental benefits of outdoor nature play are well-defined, research examining the possible benefits for children of including nature in interior play areas has been greatly limited. Playrooms can be found in a variety of sites such as in houses, daycare facilities, hospitals, libraries, and residential buildings. These spaces can be designed to provide visual imagery, engaging stimuli in its functional areas, and the texture of objects and surfaces (McCoy and Evans, 2002). Recognizing the benefits of nature play, children’s playrooms can become an analog of a natural outdoor play space by including biophilic design elements that extend nature’s restorative effects indoors (Kellert, 2008; Kjellgren and Buhrkall, 2010).

Swank and Shin (2015) conducted three case studies introducing natural materials into nature-based playrooms. Three children were observed for the benefits of nature-based play therapy. The rationale for nature-based play therapy is to take an ecological approach to counseling in order to restore psychological functioning. The three case studies of children with varying behavioral issues such as disruptive behavior and ADHD showed that nature-based play therapy contributed to displayed significant improvement in behavior. For the child with disruptive behavior, the natural playroom contained many opportunities for exploration, imaginative play, and practicing coping skills. One of the children with ADHD expressed excitement in exploring their natural indoor environment and more decision-making confidence. Another child with ADHD showed improvements in reducing negative attention-seeking behavior. They found natural features in the room that promoted their problem-solving, storytelling, and creativity. From a therapeutic perspective, playrooms are optimal indoor environments for combining play-therapy and biophilia to the child’s benefit.

Weinberger et al. (2017) surveyed child life specialists at a hospital to evaluate which elements within hospital playrooms are of importance to children. Biophilic elements were of high value to specialists. Elements of interest within these playrooms due to their influence on health outcomes and play include the presence of windows, access to natural light, natural colors, spaciousness, and nature-themed designs.

Present research about nature in indoor playrooms has primarily examined playrooms in institutions. They also do not consider the type of city setting in which these playrooms are situated. In urban settings where shared playrooms in residential buildings are more abundant, no study has quantified and assessed biophilic design within these spaces. Because children in urban cities spend more time indoors rather than natural outdoor spaces, their play areas indoors deserve more consideration. To aid in the incorporation of biophilic design of these spaces, a reliable tool is necessary to identify and evaluate biophilic elements.

Relatively little research has been done to develop a reliable coding system to identify biophilic attributes in different indoor settings. To fill this gap, McGee and Marshall-Baker developed the Biophilic Interior Design Matrix (BID-M, 2012) to aid designers and other specialists in quantifying biophilic features in interior spaces. The matrix was created as a tool to identify biophilic features and assess the presence and absence of those features in the built environment. Based on Kellert (2008) research, the matrix consists of 52 biophilic attributes categorized under six biophilic design elements. Twenty out of 72 original attributes were excluded from the matrix. This was due to the inability to analyze them visually and not being related to a space’s interior.

McGee and Marshall-Baker (2015) first tested the BID-M to assess biophilic interior design quality within hospital playrooms. The purpose was to evaluate the inclusion of biophilic elements in play areas in child healthcare centers aimed to benefit children’s health within the facility. The initial testing of the BID-M coding system includeda small sample of 24 playrooms in child healthcare centers and a smaller reliability sample of 4 playrooms. Inter-rater reliability of the BID-M was measured with only percent agreement, which is not a robust reliability statistic. Given the limitations of the study, additional research quantifying and assessing reliability of the BID-M is necessary. Recently, McGee et al. (2019) further developed the BDM, that now contains six elements and 54 attributes, and it is now called the Biophilic Interior Design Matrix (BID-M). The new matrix has been tested for adults’ everyday interior living places (i.e., a recreation space on a university campus). The usage of the matrix should be amplified for the evaluation of biophilic interior design for children’s everyday living places, the importance of biophilic characteristics in the construction of their environmental identity, their environmental knowledge and their pro-environmental behavior as adults. It is particularly important, especially at a time when a large part of the world’s population, including children, has experienced a lockdown at home due to safety measures in place during the COVID-19 pandemic, to take into account wellbeing in our homes, our daily living places. Studies such as the current study are a step forward.

The current study built upon McGee and Marshall-Baker (2015) study by evaluating the reliability of the BID-M for its use in children’s playrooms in urban residential buildings. We focused on a very particular socio-cultural context, Manhattan, a borough in New York City. Neighborhood compositional characteristics influence the access to nature in New York City (Weiss et al., 2011; Huang et al., 2020). By focusing on Manhattan, we minimized the influence of socio-cultural context.

We improved upon the original study by evaluating playrooms catered to a more generalized population of children rather than a specific subset of children who are under hospital care. We assessed a larger sample of playrooms compared to the original study for inter-rater reliability of the biophilic design matrix. This study is a first in the consideration of this type of place. The results of this study might be particularly interesting, given that these are places where children spend a lot of time.

## Materials and Methods

### Sampling Method

**FIGURE 1:**
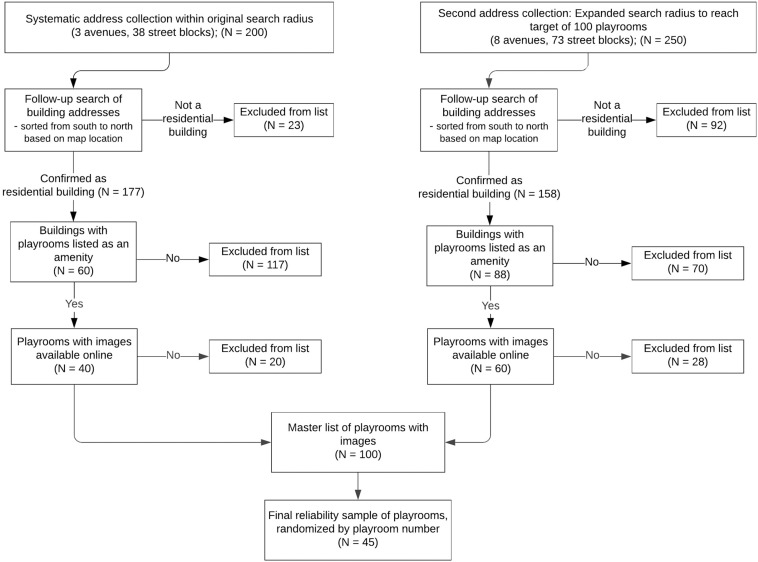
Flowchart of systematic collection of playroom images. Playroom numbering was based on their location from south to north, organized by street address in ascending order numerically and alphabetically. For our final study sample, we used a number generator to randomly select 45 playrooms from our master list of 100 playrooms.

**FIGURE 2:**
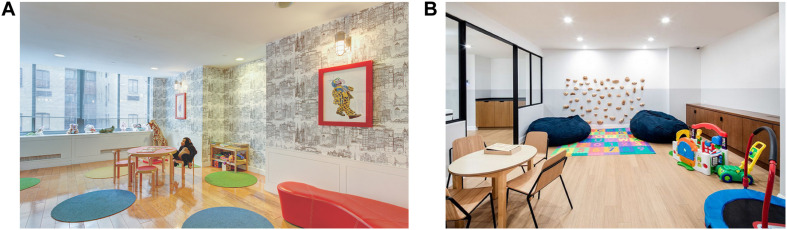
**(A)** Image of Playroom 7. Reprinted with permission. **(B)** Image of Playroom 22. Reprinted from *Oriana*. Retrieved from https://oriananyc.com /amenities/. Reprinted with permission.

**FIGURE 3:**
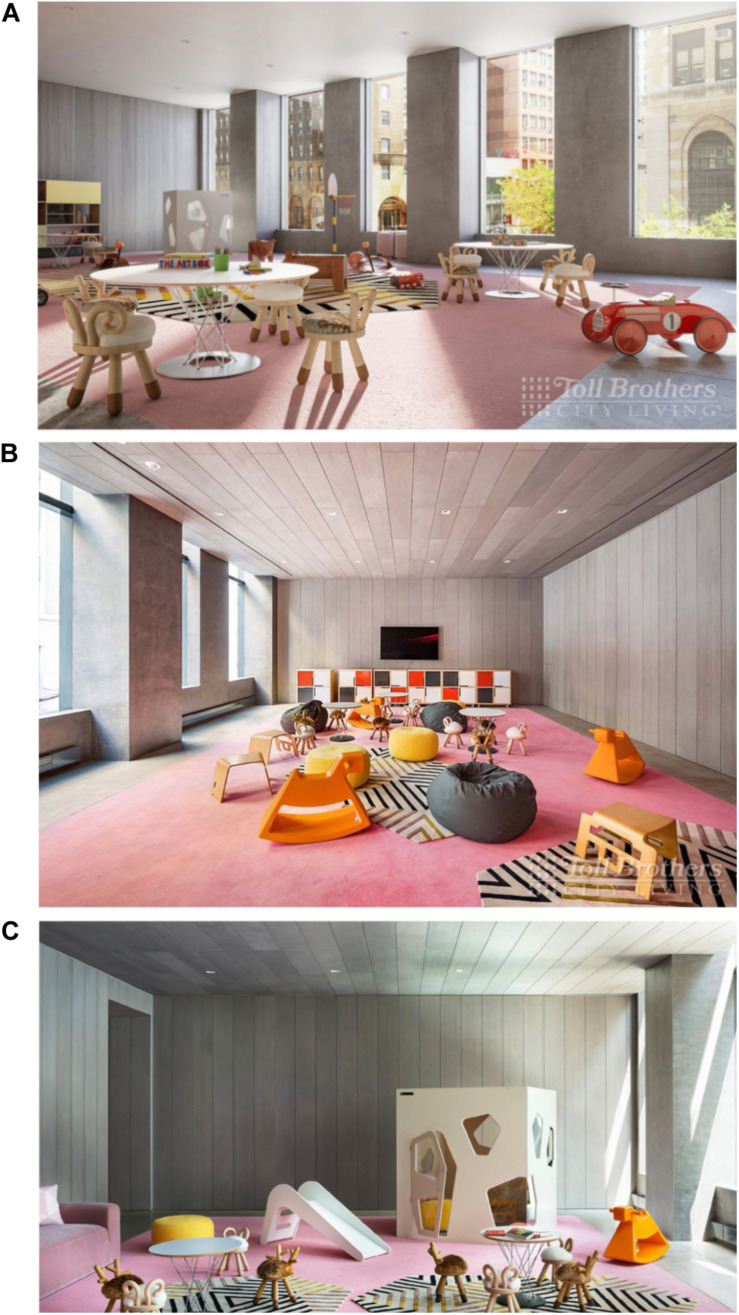
Examples of multiple images collected for one playroom. All images collected of each playroom were coded using the BID-M. **(A)** Reprinted from Joseph (n.d. a). **(B)** Reprinted from Joseph (n.d. b). **(C)** Reprinted from Joseph (n.d. c).

Figure 1 displays a flowchart of the sampling process. We conducted a systematic search of residential buildings in Manhattan with playrooms designated for children. Building searches were conducted through both in-person address collection and online map searches in the form of a grid of the east side of Manhattan. In-person searches involved the researchers walking down streets and taking down addresses. Map searches online involved the same grid search, moving block by block through an online map within our search radius and collecting addresses of residential buildings. Townhouses and single-family residences were excluded After address collection, we conducted a follow-up search to determine if the residential buildings offered a playroom area through locating an existing website listing the building’s amenities. Multiple websites were searched if initial web searches were not clear regarding the building’s amenities. All buildings with a playroom as an amenity in our search area were put into a master list of playrooms. If a building met the criterion of having a playroom as a listed amenity, photographs of the playroom needed to be available online to be included in the finalized list. Figures 2, 3 present examples of images used. As shown in Figure 3, for some playrooms, multiple online images of the same playroom were available. All available images were collected to be used for coding.

**FIGURE 4:**
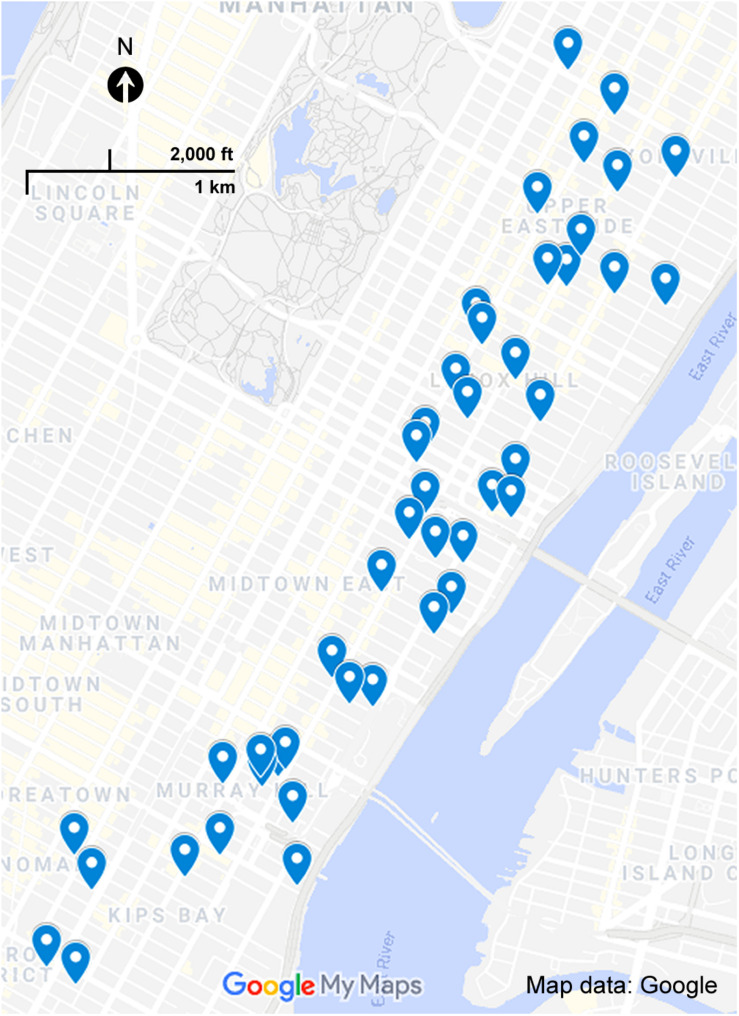
Map of Manhattan with pinned playroom locations. Google (n.d.). [Playroom Locations in Manhattan]. Retrieved April 3, 2020 from https://goo.gl/maps/wdMUjEQ6nDKk8oEe6.

Our original search radius was, from east to west, across three avenues (York Avenue to Second Avenue) and within 38 street blocks (30th Street to 68th Street). A large number of buildings in our master list with playrooms as an amenity had no photos available online. Due to inclusion criteria of needing to have an image of the playroom available online to be included in the finalized sample, 20 buildings were not used. To reach our initial target number of playrooms of 100, our search area needed to be expanded. The locations of the playrooms range 73 street blocks from south to north (22nd Street to 95th Street) and across eight avenues from east to west (York Avenue to Fifth Avenue). Our final randomized sample consisted of 45 playrooms with digital images. Figure 4 displays a map of Manhattan with the locations of all 45 playrooms. Playrooms were assigned numbers after being sorted by ascending order of street number and building address, starting south to north from 22nd Street to 95th Street.

### Measures

#### The Biophilic Interior Design Matrix

**FIGURE 5:**
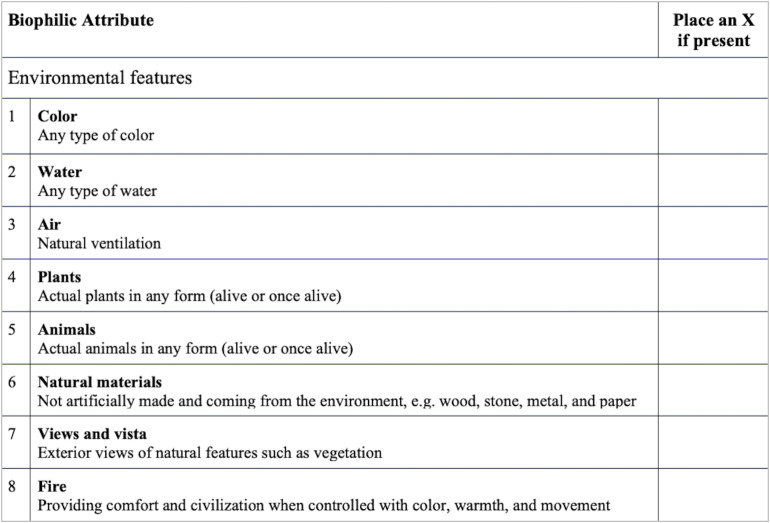
Sample of the Biophilic Interior Design Matrix (BID-M). Shown is one out of six biophilic elements and its attributes. Adapted from McGee and Marshall-Baker (2015).

The original BID-M (McGee and Marshall-Baker, 2015) included a quantitative element of scoring playrooms based on presence or absence of an attribute and a photo-ethnographic component to describe the presence of each attribute both verbally and photographically. As shown in Figure 5, our adaptation of the BID-M solely focuses on the quantitative component. Using the BID-M protocol and all images of the space, playrooms were scored from 0 to 52 for biophilic design attributes in the room. The number of images analyzed per playroom ranged from 1 to 4, with a mean of 1 picture for each playroom and a standard deviation of 0.6878. Even though the setting differs from that of the study by McGee and Marshall-Baker (2015), we examined to what extent the BID-M is reliable to use in different venues for children such as residential playrooms and to what extent it can be refined for the specific context we are observing. Therefore, no changes were made to the original matrix. The attributes and element categorization used for the matrix are based on those by Kellert (2008). Specifically, the BID-M includes environment features, natural shapes and forms, natural patterns and processes, light and space, place-based relationships, and human-based relationships. Within these six elements are a wide range of attributes such as air (natural ventilation), plants (alive or once alive), animal motifs (representations of animals or animal forms) shapes resisting straight lines, patterned wholes (e.g., tiled floors), integration of parts to wholes (e.g., individual wooden planks making up a wooden floor), natural light, filtered and diffused light, spaciousness, spatial variability (visual variability in light and overall definition of the space such as ceiling height, room widths, etc.), geographic connection to place (the use of local features and the selection of views), and prospect and refuge (a space with areas where children can survey the space and can isolate).

One point was received if an attribute was present, and no points were received if an attribute was absent. A higher score indicated the presence of many biophilic design attributes in the room, whereas a low score indicated that many attributes were absent in the room. For data analysis, *present* attributes were coded as a 1 and attributes that were *absent* were coded as a 0. For each playroom, a mean score was calculated across four raters. The raters were three undergraduate psychology students and the researcher, all with no background in interior design or architecture.

#### Inter-Rater Reliability

To measure reliability across four raters of the overall design matrix and the 52 matrix items, we calculated percent agreement and Randolph’s free-marginal multirater kappa (Randolph, 2005). Free-marginal multirater kappa is an alternative to Fleiss’ multirater kappa (Fleiss, 1971). Fleiss’ multirater kappa is a fixed-marginal reliability statistic used when there is a predetermination of how many items multiple raters distribute or code into each nominal category. A limitation of fixed-marginal multirater kappa being used in free-marginal cases to measure agreement is that the varying marginal distributions in each case greatly affect the value of kappa even when overall agreement is constant. Free-marginal multirater kappa is not held to the restriction of fixed marginal item distributions. This multirater kappa is the suitable reliability statistic for our study design as there is no *a priori* determination of how many matrix items are distributed into the categories of presence or absence for each coded playroom.

Two initial rounds of reliability testing were conducted for raters to gain familiarity with the matrix. Each round of reliability testing consisted of six different playrooms with 12 playrooms total out of the 45-room sample. All playrooms were independently coded using the matrix by all four raters. Discussions about coding disagreements and clarity of definitions were had after each assessment before analyzing the larger reliability sample of 33 playrooms. The two rounds of reliability testing of the overall design matrix had a kappa = 0.60 (80.13%) and kappa = 0.64 (81.94%), respectively. Regarding the strength of kappa, between 0.41 and 0.60 is moderate agreement, 0.61 and 0.80 is good agreement, and 0.81 and 1.00 is excellent agreement (Landis and Koch, 1977). Inter-rater reliability of the overall design matrix and individual items within the matrix was calculated for the remaining 33 playrooms out of our 45-room sample.

## Results

### Mean Design Matrix Scores

**FIGURE 6:**
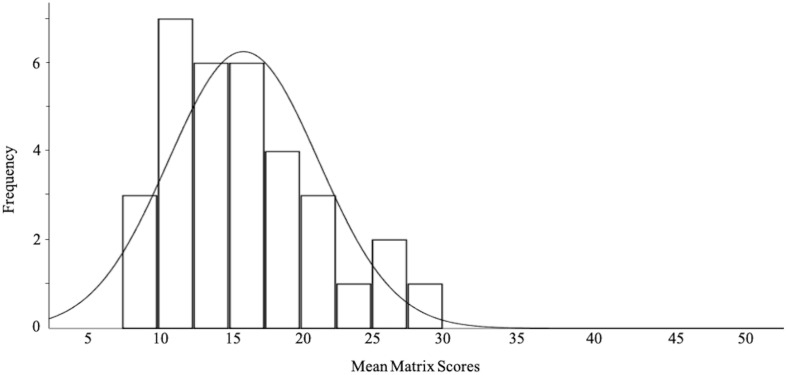
Histogram of mean matrix scores of all 33 playrooms. A majority of matrix scores fell below the sample mean of 16.015 out of a maximum possible score of 52. Skewness = 0.838.

Figure 6 presents a histogram of the mean matrix scores for the larger reliability sample of 33 playrooms. The average of all total BID-M scores in our sample was 16.015 out of 52 (*SD* = 5.25). The range of scores was 20.75, with the lowest score being 8.75 out of 52 and the highest score being 29.50 out of 52. The data is moderately skewed left due to the majority of the matrix scores falling below the mean of 16.015.

### Inter-Rater Reliability of Overall Design Matrix and Matrix Items

Percent agreement and free-marginal multirater kappa (multirater κ_*free*_) were calculated using our larger reliability sample of 33 playrooms to measure the reliability of the overall design matrix as well as the 52 biophilic design attributes within the matrix.

As shown in Table 2, results of inter-rater reliability testing for 33 playrooms showed that the overall design matrix showed good agreement with a kappa of 0.65 and an average percent agreement of 82.5%. Using a two-sided 95% confidence interval, multirater kappa showed that there was good agreement across the raters’ matrix scores, multirater κ_*free*_ = 0.65 (*SE* = 0.18), 95% CI [0.59, 0.72], with an average percent agreement of 82.5%. Table 2 presents 31 out of 52 items within the design matrix that had an acceptable kappa value of 0.61 in boldface. 21 items (40.4%) yielded kappa values below 0.61. All six elements contained at least one item with low reliability (multirater κ_*free*_ < 0.61). The items with low reliability were: air (0.54), tree and columnar supports (0.44), shells and spirals (0.53), egg, oval and tubular forms (0.20), arches, vaults, and domes (0.37), shapes resisting straight lines (0.10), simulation of natural features/biomorphy (0.37), sensory variability/information richness (0.46), patterned wholes (0.57), bounded spaces (0.49), integration of parts to wholes (0.51), complementary contrasts (0.56), fractals (0.59), light and shadow (0.29), reflected light (0.25), spatial variability (0.60), space as shape and form (0.60), spatial harmony (0.36), geographic connection to place (0.40), ecological connection to place (0.54), and curiosity and enticement (0.16). As for the elements that held the majority of the 21 items with low reliability, items within “natural shapes and forms” accounted for 6 out of 21 items (28.6%) and items within “natural patterns and processes” accounted for 6 out of 21 items (28.6%).

**TABLE 2:** Results of inter-rater reliability testing of all 52 matrix items for 33 playrooms.

**Biophilic attributes**	**κ_*free*_**	**Percent agreement (%)**	**95% CI**
**Environmental features**			
Color	**1.00**	100.0	[1.00, 1.00]
Water	**0.84**	91.9	[0.70, 0.97]
Air	0.54	76.8	[0.34, 0.73]
Plants	**0.94**	97.0	[0.86, 1.00]
Animals	**0.88**	93.9	[0.77, 0.99]
Natural materials	**0.74**	86.9	[0.57, 0.90]
Views and vistas	**0.87**	93.4	[0.74, 0.99]
Fire	**1.00**	100.0	[1.00, 1.00]
**Natural shapes and forms**			
Botanical motifs	**0.61**	80.3	[0.42, 0.79]
Tree and columnar supports	0.44	72.2	[0.25, 0.63]
Animal motifs	**0.68**	83.8	[0.51, 0.85]
Shells and spirals	0.53	76.3	[0.34, 0.71]
Egg, oval, and tubular forms	0.20	60.1	[0.03, 0.38]
Arches, vaults, and domes	0.37	70.7	[0.18, 056]
Shapes resisting straight lines	0.10	55.1	[−0.02, 0.22]
Simulation of natural features/biomorphy	0.37	68.7	[0.18, 0.56]
Geomorphology	**0.85**	92.4	[0.72, 0.97]
Biomimicry	**0.65**	82.3	[0.47, 0.82]
**Natural patterns and processes**			
Sensory variability/Information richness	0.46	73.2	[0.28, 0.65]
Age, change, and the patina of time	**0.97**	98.5	[0.91, 1.00]
Central focal point	**0.66**	82.8	[0.49, 0.83]
Patterned wholes	0.57	78.3	[0.38, 0.76]
Bounded spaces	0.49	74.8	[0.30, 0.69]
Transitional spaces	**0.78**	88.9	[0.63, 0.93]
Linked series and chains	**0.77**	88.4	[0.61, 0.92]
Integrations of parts to wholes	0.51	75.3	[0.31, 0.70]
Complementary contrasts	0.56	77.8	[0.37, 0.74]
Dynamic balance and tension	**0.72**	85.9	[0.56, 0.88]
Fractals	0.59	79.3	[0.41, 0.77]
Hierarchically organized ratios and scales	**0.94**	97.0	[0.86, 1.00]
**Light and space**			
Natural light	**0.63**	81.3	[0.45, 0.80]
Filtered and diffused light	**0.91**	95.4	[0.81, 1.00]
Light and shadow	0.29	64.7	[0.12, 0.47]
Reflected light	0.25	62.6	[0.08, 0.43]
Light pools	**0.91**	95.5	[0.81, 1.00]
Warm light	**0.66**	82.8	[0.47, 0.84]
Light as shape and form	**0.78**	88.9	[0.63, 0.93]
Spaciousness	**0.73**	86.4	[0.57, 0.88]
Spatial variability	0.60	79.8	[0.41, 0.78]
Space as shape and form	0.60	79.8	[0.42, 0.77]
Spatial harmony	0.36	68.2	[0.18, 0.54]
Inside-outside space	**0.97**	98.5	[0.91, 1.00]
**Place-based relationships**			
Geographic connection to place	0.40	70.2	[0.21, 0.60]
Historic connection to place	**0.85**	92.4	[0.72, 0.97]
Ecological connection to place	0.54	76.8	[0.34, 0.73]
Cultural connection to place	**0.71**	85.4	[0.54, 0.87]
Indigenous materials	**1.00**	100.0	[1.00, 1.00]
Landscape orientation/landscape features	**0.62**	80.8	[0.44, 0.79]
**Human-nature relationships**			
Prospect and refuge	**0.86**	92.9	[0.73, 0.99]
Order and complexity	**0.63**	81.3	[0.45, 0.80]
Curiosity and enticement	0.16	58.1	[−0.01, 0.33]
Change and metamorphosis	**0.79**	89.4	[0.65, 0.93]
Total agreement	0.65	82.5	[0.59, 0.72]

*31 matrix items with kappa values greater than or equal to 0.61 are in boldface.*

### Schematic Model of Biophilic Playroom

**FIGURE 7:**
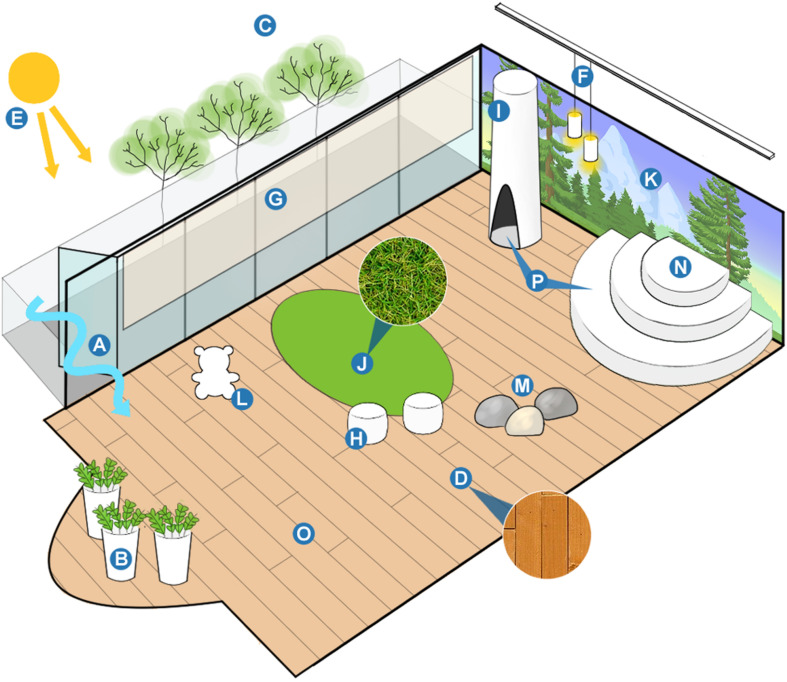
Basic schematic model of biophilic playroom based on matrix attributes present in our sample of playrooms. Attributes included were present in rooms that scored highest in our sample and are attributes noted to be generally important to biophilic design in current literature. One object can fulfill more than one attribute. **(A)** Air. Operable windows and doors that allow natural airflow. Inside-outside space. Interior spaces connected to exterior environments (e.g., porches, foyers, and gardens). **(B)** Plants. Alive or once alive. **(C)** Views and vistas. Views of exterior vegetation from the interior. Age, change, and the patina of time. Change and metamorphosis. An example of both attributes is the view of vegetation that change, grow, and bloom through the seasons. **(D)** Natural materials. The use of naturally found materials to construct objects in the space (e.g., wood, stone, metal, and paper, etc.). Integration of parts to wholes. An example of this attribute is the use of individual wooden planks to make up a hardwood floor, or more conceptual such as objects being used to create an overall cohesive theme in the room. Images are Creative Commons. **(E)** Natural light. *Warm light.* **(F)** Warm light. Egg, oval, and tubular forms. **(G)** Filtered and diffused light. Modulated daylight to reduce glare, such as through the use of blinds and shades. **(H)** Egg, oval, and tubular forms. Shapes resisting straight lines. In many playrooms, these attributes were seen in the form of tubular/circular seating, light fixtures, and other décor in the room. **(I)** Botanical motifs. Tree and columnar supports. Biomimicry. **(J)** Sensory variability. An example is the variability between the texture of hardwood floors and a carpet meant to simulate grass. Additionally, varying light sources, textures, and visuals contribute to this attribute. **(K)** Botanical motifs. Shapes, forms, patterns, and images of plants and vegetation. Image is Creative Commons. **(L)** Animal motifs. Representations of animals. Many rooms displayed this attribute in the form of toy animals as well as in the form of stylized furniture. Image is Creative Commons. **(M)** Geomorphology: Playrooms contained either imagery of rock formations or toy rocks. **(N)** Arches, vaults, and domes. Shapes resisting straight lines. A climbing structure, also in the shape of an arch, meant to represent a vault or natural cliff. **(O)** Spaciousness. Open space for children to engage in active play. Spatial variability. Variability in space shape can be done through the use of columns, along with varied wall widths and ceiling heights. **(P)** Prospect and refuge. For human-based relationships, both characteristics of prospect and refuge need to be present. This can be fulfilled by having an area where children can go and hide for solace, and an area with the ability to survey the room.

In Figure 7, we represented a basic schematic model of a biophilic playroom based on matrix attributes present in our sample of playrooms. Attributes included in the model were present in rooms that scored highest in our sample or are attributes noted to be generally important to biophilic design in current literature. One object can fulfill multiple attributes. As it is possible to see, the ideal biophilic playroom may be characterized by access to natural and warm light, views of exterior vegetation, the use of natural materials (e.g., wood, stone, metal, and paper), operable windows and doors that allow natural air ventilation, interior plants, botanical imagery and animal motifs within the room, representations of rocks or rock formations, open space, and shapes found in nature such as circles, ovals, tubular forms, sinuous or flowing lines, and arches. More abstract concepts presented in the model include variability in sensory input and in the shape of the space itself, the ability to view the passage of time through seasonal changes to exterior vegetation, individual objects that together comprise either a larger structure or cohesive theme in the room, and the fostering of human-based relationships through characteristics that allow for a meaningful connection to the space such as prospect and refuge, and change and metamorphosis.

## Discussion

The purpose of this study was to evaluate the reliability of the BID-M developed by McGee and Marshall-Baker (2015) for its applicability to urban residential playrooms. No study has evaluated the measure in playrooms outside of institutional buildings (such as children’s healthcare facilities) or for its suitability in urban interior spaces. Children experience substantial psychological benefits when engaging in outdoor play in natural environments (Fjørtoft, 2001; Chawla, 2007; McCurdy et al., 2010). Only recently have researchers studied biophilic interior spaces and present findings showing the importance of natural features in playrooms on cognitive development, social-emotional development, and overall wellbeing (Swank and Shin, 2015; Weinberger et al., 2017). In order to later assess the biophilic quality in urban residential playrooms and the psychological effects that children experience in a low biophilic playroom and a high biophilic playroom, assessing the methodological soundness of a measure that identifies and quantifies biophilic design features in these spaces is needed.

We focused on a very particular socio-cultural context, Manhattan. Neighborhood compositional characteristics influence the access to nature in New York City (Weiss et al., 2011; Huang et al., 2020). By focusing on this specific area of New York City, we minimized the influence of socio-cultural context. We improved on the original study by evaluating a larger sample of rooms along with a larger reliability sample. We additionally evaluated playrooms designated for a more general population of children as the original study tested the BID-M in hospital playrooms.

Even though the results of inter-rater reliability testing of the overall design matrix in our study showed good agreement, several matrix items showed low reliability. A possible explanation for many scores falling below the sample mean, and low inter-rater reliability of matrix attributes might be that the original BID-M (McGee and Marshall-Baker, 2015) was not designed to quantify biophilic features in urban playrooms for children. There is a need to modify the coding system to remove or clarify items that reached lower reliability depending on their suitability in children’s playrooms and their feasibility in urban interior spaces.

Another reason for low reliability of attributes may be the varying complexity of the coding system. A combination of more visually objective attributes (e.g., air; tree and columnar supports; shells and spirals; egg, oval and tubular forms; arches, vaults, and domes; shapes resisting straight lines; simulation of natural features/biomorphy; patterned wholes; bounded spaces; integration of parts to wholes, complementary contrasts; fractals; light and shadow; reflective light) and subjective attributes (e.g., sensory variability, spatial variability; space as shape and form; spatial harmony; geographic connection to place; ecological connection to place; curiosity and enticement) yielded low reliability. Although these visually objective and subjective attributes are categorically different, both were challenging to conceptualize within playrooms. This was due to the lack of operational definitions or visual references for how these features found in nature translate to interior spaces. All four raters in the present study do not have a background in interior design or architecture unlike the raters in McGee and Marshall-Baker (2015) study. Solely relying on the verbal description of attributes allowed room for subjective interpretation of what the presence of these attributes in the built environment looks like. For example, discrepancies lied in whether the presence of arches should be coded for if it was not present in a natural semi-circle shape, i.e., not counting closed or rectangular arches. Additionally, varying perception of a photograph allows for disagreements. Shapes resisting straight lines had a discrepancy of if more sinuous and flowing lines should only be counted, or if arches as well as circles should be coded. Disagreements in coding light and shadow and reflected light may be attributed to the degree of their presence.

We looked into attributes that showed consistently high agreement as to why that may be the case. Of the 52 BID-M attributes, those were color, water, plants, animals, natural materials, views and vistas, fire, animal motifs, and age, change, and the patina of time, hierarchically organized ratios and scales, spaciousness, and prospect and refuge. For the attributes within environmental features (color; water; plants; animals; natural materials; views and vistas; and fire), this finding is unsurprising because the definitions of these attributes and their presentation in urban interior spaces is well-defined in existing research (Woolley and Lowe, 2013; An et al., 2016). High agreement in some attributes can be attributed to flaws within the coding system. For color, the definition in the BID-M is: “any type of color” present in the room. Due to this classification, color as an attribute was counted for every single playroom, even in rooms with primarily black, white, and gray colors and small objects with color. The color palette of nature, one that primarily focuses on green tones and colors of natural landscapes, has a significant influence on our connection to natural environments and on the restorative quality of nature (Browning et al., 2014). If the degree of the presence of colors and tones primarily found in nature were the attribute criteria, different results may emerge in future uses of the coding system.

We found that in the context of urban residential buildings, some attributes had 100% percent agreement simply because they were all noted as absent in all 45 playrooms (animals alive or once alive; plants alive or once alive; fire; indigenous materials; age, change, and the patina of time; hierarchically organized ratios and scales). While plants and vegetation are found to be important natural inclusions to biophilic spaces and to nature play (Woolley and Lowe, 2013; An et al., 2016; Hähn et al., 2020), features such as alive or once alive animals, fireplaces, and actively aging natural materials may not be feasible to include in an interior space in urban contexts or in playrooms. Therefore, the absence of these attributes may indicate that they do not apply fully to urban interior spaces, particularly children’s playrooms.

One of Kellert (2008) biophilic elements that we suggest for modification to be better applicable to urban interior spaces is place-based relationships. Discussing the connection-to-place attributes (geographic connection to place, historic connection to place, ecological connection to place, and cultural connection to place) highlighted confusion with these codes in the context of an urban city. Disagreements initially lied in what that “place” is: New York City and its landscape (e.g., views or murals of miscellaneous buildings and depictions of iconic buildings or natural environments). All raters initially came to agreement to code based on the setting in which the building is situated: an urban city. “Placed-based relationships” are not supported by existing research when the “place” in which the interior space is built is inherently not biophilic. Multiple studies support that stimuli in urban environments are more cognitively taxing than natural environments (Ulrich et al., 1991; Berto, 2005; Berman et al., 2008; Bratman et al., 2015; Zijlema et al., 2018) and do not positively influence child development (Preuß et al., 2019). Russ et al. (2015) posited that development of a “connection to place” with the urban environment is better cultivated through direct exposure outdoors. This suggests that place-based relationships cannot be fostered within the interior at all. Therefore, introducing attributes related to visual stimuli of the urban built environment into the space does not seem to be a contributor to biophilic quality. A coding system that takes into account the urban context would need to modify this element and its attributes to specifically assess the presence of connection to natural environments.

Descriptions of attributes should be supported by examples of how they can be found in playrooms and why they are beneficial in the context of nature play. Similar to Kellert’s biophilic design attributes, Woolley and Lowe (2013) identified that features such as landforms and natural materials provide ample play opportunities in natural environments. Sensory variability provided by differing surface materials and texture along with variability in trees and plants provide sensory stimulation that encourages interaction with nature. With regards to spatial design, spatial variability, spaciousness, and spaces with boundaries are identified as structural features that allow for exploration and free movement during play (Woolley and Lowe, 2013; Park and Lee, 2019). Besides open spaces, children benefit from places of refuge in the form of hiding places both for play purposes and for a sense of security (Park and Lee, 2019). Existing research relevant to the demographic that is engaging with these playrooms can allow for more consistent coding of attributes.

A noted limitation is that we did not photograph these spaces ourselves nor did we physically observe them due to the COVID-19 pandemic. Online images of these rooms do not provide a full view of what the room in its entirety looks like. Photographs can be angled, rendered, and edited in ways that overly enhance the image quality or diminish it. Attributes related to light and space may also be affected by the use of online images depending on the quality and angle of the image. Another example of limitations due to photograph quality is for the attribute of air, which yielded low reliability across the four raters. To code for “air,” operable windows needed to be present in the room. Photographs needed to be carefully observed for signs of an operable window such as latches. Because of varying image quality and angles of the photograph, these particular features were missed by some raters, causing inconsistent coding. The nature of the online images collected influences matrix scoring in that attributes in one image were not present in another image of the space. Extensive web searches were conducted so that all available images of a playroom could be collected in order to provide the most possible holistic observation.

A second limitation is the use of a binary quantitative measure. While we used the BID-M as is, coding playrooms only based on presence and absence is not the robust method of assessing biophilic design. Discussions regarding coding disagreements emphasized that some raters may not have coded for an attribute to do it being small or indiscernible that it should not be coded (e.g., a very small toy containing a spiral would have the “shells and spirals” attribute coded as *present*; a singular area where the width of the room varies having the “spatial variability” or “space as shape and form” attributes be coded as *present*). Future modifications of the BID-M for use in urban playrooms call for a Likert scale quantitative measure of each attribute. With a wider range of ratings, future reliability testing may prove to be more meaningful, as there is more strength in two raters both coding an attribute as a 4 on a 5-point Likert scale versus simply presence and absence.

Another limitation of the study is that we used multiple online images of the same playroom for coding. However, we did not use a standardized protocol that called for the use of the same number of images per playroom. This could be a bias to the description of the place. Specifically, it is likely that playrooms with more pictures would have been more likely to get higher scores because more information about present attributes were available online.

In this study, we focused on a very particular socio-cultural context, Manhattan. Our results call for new studies on children playrooms in other socio-cultural contexts. It might be of interest, for instance, to compare our results with a study in a where nature is highly accessible and where the culture of the inhabitants is to spend most of the time outdoors. Linking biophilic design to children’s wellbeing might inform a set of strategies that can enhance the quality of life of people living in this specific area. In a future study, we would like to consider socio-cultural context as a possible independent variable which might influence the presence of biophilic design in playrooms. To achieve this aim we will consider different neighborhoods and a wider set of playrooms.

## Conclusion

These proposed matrix modifications open new avenues for a coding system designated for interior play spaces in urban contexts, one that takes into account how children in cities experience nature differently. Because of the deficit in natural outdoor spaces in these cities, children are not adequately getting the necessary exposure to nature needed for healthy development (Louv, 2008). To assess the biophilic properties of a playroom fit for urban children, certain biophilic attributes present in an outdoor environment that are conducive to nature play and development should be considered.

Existing research can supplement the BID-M to develop a biophilic interior design checklist for attributes conducive to nature play in urban interior spaces. This would allow for easier identification and assessment of biophilic features of playrooms that may transform them into a natural environment with opportunities for exploration, visual stimulation, and cognitive development. Attributes that are not feasible in urban interior spaces should be removed in future iterations of this matrix. Additionally, the binary categorization of the matrix should be revised to a scale to better assess the quality. Assessing biophilic quality on a binary presence and absence categorization is inadequate in evaluating the scale at which some attributes are present. Another modification is to move beyond visual characteristics to assess biophilic quality. Nature is a multi-sensory experience, in both natural green spaces and urban green spaces. For example, olfactory stimuli and auditory stimuli such as bird songs in urban parks contribute to nature’s restorative effect and may reduce stress (Hedblom et al., 2019). While the measure used in this study focuses on visual stimuli to assess biophilic quality, future matrix modifications can account for various multi-sensory characteristics that influence children’s holistic experiences in natural playrooms. Future studies may also use this refined measure to investigate the effects of varying biophilic design quality of playrooms on aspects of cognitive development in children. Not only interior designers and urban planners, but parents and caregivers in urban settings can use this measure to design a playroom in a practical way that still provides the restorative effect that nature offers. Goals for this tool are that it should (1) apply to interior spaces in urban contexts, (2) take into account current research regarding how children experience the natural environment through play, and (3) have increased usability across users of different knowledge backgrounds.

## Data Availability Statement

The original contributions presented in the study are included in the article/Supplementary Material, further inquiries can be directed to the corresponding authors.

## Author Contributions

TS conceptualized and designed the research. EM, BB, AC, and AT defined the methodology and wrote the original draft with the contribution of all authors. EM and BB analyzed the data with the supervision of RF. EM designed the figures with the supervision of TS and RF. RF and TS reviewed the manuscript. All authors discussed the data, edited the manuscript, and approved the manuscript.

## Conflict of Interest

The authors declare that the research was conducted in the absence of any commercial or financial relationships that could be construed as a potential conflict of interest.
