# Supplementary material for: Testing Reliability of Biophilic Design Matrix Within Urban Residential Playrooms
Source: Front Psychol. 2020 Dec 9;11:570099. doi: 10.3389/fpsyg.2020.570099 (PMC7756148; doi:10.3389/fpsyg.2020.570099)
Supplement: Supplementary file 1 [file Table_1.DOCX]

**Supplementary Table 1.** Mean BID-M scores, standard deviations, and range of scores from main sample of 33 playrooms. All matrix scores are out of a possible maximum score of 52. The matrix scores for each playroom presented are the mean of matrix scores given by four raters.

| Playroom # | *M* | *SD* | Range |
| --- | --- | --- | --- |
| 1 | 24.75 | 3.304 | 21-29 |
| 2 | 15.75 | 3.594 | 13-21 |
| 3 | 21.5 | 1.732 | 19-23 |
| 4 | 22.25 | 2.062 | 20-25 |
| 5 | 17.75 | 3.686 | 13-22 |
| 6 | 14.25 | 4.924 | 8-20 |
| 8 | 16 | 1.414 | 15-18 |
| 11 | 14.75 | 2.217 | 13-18 |
| 12 | 15.75 | 3.594 | 13-21 |
| 14 | 10.5 | 2.380 | 7-12 |
| 15 | 18 | 2.160 | 16-21 |
| 16 | 10.25 | 2.754 | 7-13 |
| 21 | 11.5 | 2.646 | 9-15 |
| 22 | 9.5 | 1.000 | 9-11 |
| 24 | 12.25 | 3.304 | 8-16 |
| 25 | 12.25 | 2.062 | 12-15 |
| 26 | 11.5 | 1.291 | 10-13 |
| 27 | 12 | 4.082 | 9-18 |
| 28 | 16.25 | 4.425 | 12-21 |
| 29 | 13.75 | 4.349 | 8-18 |
| 35 | 13 | 3.367 | 11-18 |
| 36 | 25 | 4.320 | 21-31 |
| 37 | 12.5 | 4.435 | 6-16 |
| 38 | 9.25 | 3.403 | 6-14 |
| 39 | 15.75 | 4.349 | 12-22 |
| 40 | 14 | 4.546 | 9-20 |
| 45 | 26 | 1.633 | 24-28 |
| 47 | 19.75 | 5.315 | 13-26 |
| 48 | 18.5 | 4.796 | 12-23 |
| 49 | 15.25 | 4.787 | 11-22 |
| 50 | 8.75 | 2.630 | 5-11 |
| 54 | 29.5 | 3.873 | 24-33 |
| 59 | 20.75 | 4.349 | 15-25 |
| Average | 16.015 | 3.297 |  |
